# Comparative genomic analysis between newly sequenced *Brucella suis* Vaccine Strain S2 and the Virulent *Brucella suis* Strain 1330

**DOI:** 10.1186/s12864-016-3076-5

**Published:** 2016-09-20

**Authors:** Dong-dong Di, Hai Jiang, Li-li Tian, Jing-li Kang, Wen Zhang, Xin-ping Yi, Feng Ye, Qi Zhong, Bo Ni, You-yu He, Lin Xia, Yao Yu, Bu-yun Cui, Xiang Mao, Wei-xing Fan

**Affiliations:** 1 Laboratory of Zoonoses, China Animal Health and Epidemiology Center, Qingdao, Shandong China; 2 Chinese Academy of Agricultural Sciences, Shanghai Veterinary Research Institute, Shanghai, China; 3 State Key Laboratory for Infectious Disease Prevention and Control, Collaborative Innovation Center for Diagnosis and Treatment of Infectious Diseases, National Institute for Communicable Disease Control and Prevention, Beijing, China; 4 Xinjiang Academy of Animal Science, Institute of Veterinary Research, Urumuqi, Xinjiang China; 5 ZhongXin Biotechology Shanghai Co, Ltd. 12F, Building 1, 100 Qinzhou Road, Shanghai, China

**Keywords:** *Brucella suis* vaccine strain S2, *Brucella suis* virulent strain 1330, Genome sequence, Comparative genomic analysis

## Abstract

**Background:**

Brucellosis is a bacterial disease caused by *Brucella* infection. In the late fifties, *Brucella suis* vaccine strain S2 with reduced virulence was obtained by serial transfer of a virulent *B. suis* biovar 1 strain in China. It has been widely used for vaccination in China since 1971. Until now, the mechanisms underlie virulence attenuation of S2 are still unknown.

**Results:**

In this paper, the whole genome sequencing of S2 was carried out by Illumina Hiseq2000 sequencing method. We further performed the comparative genomic analysis to find out the differences between S2 and the virulent *Brucella suis* strain 1330. We found premature stops in outer membrane autotransporter *omaA* and *eryD* genes. Single mutations were found in phosphatidylcholine synthase, phosphorglucosamine mutase, pyruvate kinase and FliF, which have been reported to be related to the virulence of *Brucella* or other bacteria. Of the other different proteins between S2 and 1330, such as Omp2b, periplasmic sugar-binding protein, and oligopeptide ABC transporter, no definitive implications related to bacterial virulence were found, which await further investigation.

**Conclusions:**

The data presented here provided the rational basis for designing *Brucella* vaccines that could be used in other strains.

**Electronic supplementary material:**

The online version of this article (doi:10.1186/s12864-016-3076-5) contains supplementary material, which is available to authorized users.

## Background

Brucellosis is a worldwide zoonotic disease caused by the infection of *Brucella* species, a Gram-negative and facultative intracellular bacterium. Brucellosis can be acquired by direct contact with infected animals including cattle, sheep, goats and swine. *Brucella* infects approximately 500,000 humans worldwide annually according to the World Health Organization (WHO) [1]. The infection can cause reproductive failure in food animals and even the loss of human productivity. The clinical symptoms of *Brucella* infection include undulant fever, abortion, asthenia, endocarditis, and encephalitis. Brucellosis in food animals could be controlled by vaccination, while human brucellosis can be treated with antibiotics.

Currently, there are ten recognized *Brucella* species based on their host preference and phenotypic differences: *B. melitensis* (goats), *B. abortus* (cattle), *B. suis* (swine), *B. canis* (dogs), *B. ovis* (sheep), *B. neotomae* (desert mice), *B. ceti* (cetaceans), *B. pinnipedialis* (pinnipeds), *B. microti* (common voles) and *B. inopinata* (unknown) [2]. The genome of *B. melitensis* strain 16M is the first one to be sequenced [3], followed by *B. suis* strain 1330 [4, 5] and *B. abortus* strain 2308 and 9–941 [6]. New *Brucella* genome sequences are increasing, and at least 39 *Brucella* strains have been sequenced. These genomic resources can be served for identifying *Brucella* gene variability that associated with their host preference, pathogenesis, and virulence.

*B. suis* strain 1330 (hereafter referred to as 1330) is a swine isolate and used as the standard reference strain for *B. suis* biovar 1 [7]. According to phylogenetic analysis of 10 *Brucella* strains, *B. suis* has a broader host specificity without any identified species-specific markers [8].

The *B. suis* strain S2 is isolated from swine fetus by the Institute of Inspection for Veterinary Medicine in 1952 in China. It possesses all of the characteristics of *B. suis* biovar 1 and a smooth colonial morphology [9]. After serial transfer on media for years, *B. suis* strain S2 is naturally attenuated to a vaccine strain designated as *B. suis* vaccine “strain 2” or S2 (hereafter referred to as S2). The avirulent property of S2 is stable when inoculated into susceptible animals, and it does not cause the abortion of pregnant female [9]. Therefore, it has been widely used in China since 1971 to vaccinate sheep and goats [9]. The vaccine is administered through drinking water, a dose of 10 billion bacteria provides the protection for 2–3 years. In the middle of 1980s, S2 is introduced to the other countries [10]. Although studies suggest that the protection rate of S2 is less than that of *B. abortus* “strain 19” or S19 (hereafter referred to as S19) and *B. melitensis* Rev. 1 (hereafter referred to as Rev. 1) [11, 12], a large-scale animal test shows that S2 can provide a satisfactory protection rate in the field [9, 13]. Based on these encouraging results, the low virulence compared with S19 and Rev.1, the low cost of production, the safety for pregnant sheep and the feasibility of administration by the oral route in cattle, pigs, sheep and goats, S2 is used successfully in China to vaccinate all target species against brucellosis by administration through drinking water [9–12]. In mice, S2 has low residual virulence and less persistence time compared with S19 and Rev.1. It provides short-time protection against virulence *B. melitensis* challenge compared with the other two vaccines [14]. However, it does not show significant protection against *B. melitensis* 53H38 challenge when tested in lambs [11].

The underlying molecular and physiological mechanisms of the low virulence of S2 are not well understood so far. In this study, we performed the paired-end sequencing combined with Sanger sequencing method to determine the complete genome sequences of S2. We further identified the different genes between S2 and 1330 by genome comparison. Several putative factors were identified as the candidate genes that might be related to the virulence attenuation of S2. Further investigation of these target genes will be important for designing better vaccines to provide efficient control for brucellosis.

## Results and discussion

### Genome sequence properties

**Fig. 1:**
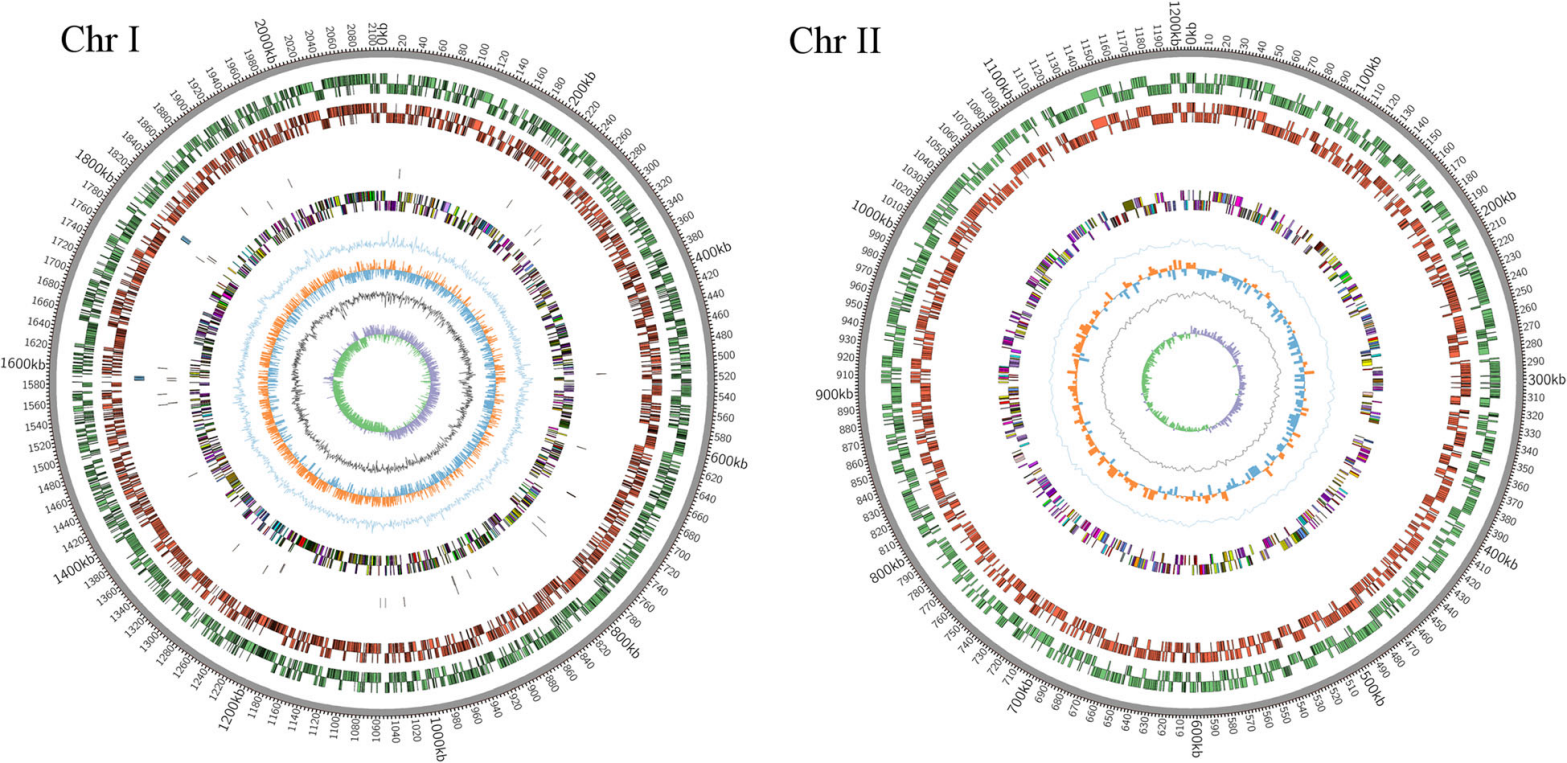
Circular representation of *Brucella suis* strain S2 chromosome I and II. Based on ProK, NR, CDD, COG, tRNA and rRNA results, two circular diagrams of chromosomes are drawn. The concentric circles show (reading outwards): GC skew, GC content, AT skew, AT content, COG classification of proteins on reverse and forward strand, tRNA, rRNA, CDS on reverse and forward strand, ORFs on three frames in reverse and forward strand

**Fig. 2:**
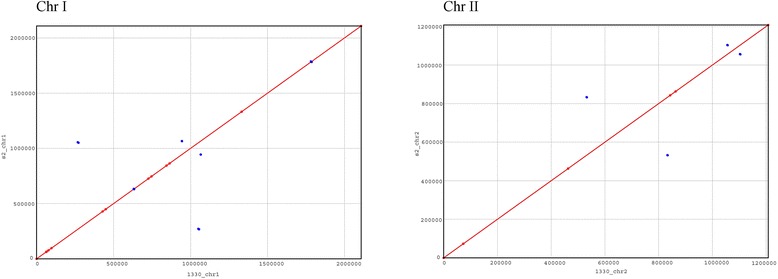
Comparative genomic analysis of the *Brucella suis* strain S2 with virulent strain 1330. The colinear analysis was carried out by Mummer. The two chromosomes of S2 were compared separately with the corresponding chromosomes of 1330. The red showed the forward sequences, while the blue represented the reverse sequence. The X-axis is the 1330 genomic sequence, Y-axis is the S2 genomic sequence. The colinear analysis showed that two chromosomes are almost identical; no large insertion/deletions were found

The S2 genome was comprised of two circular chromosomes (Table 1). One chromosome was 2,107,842 bp and the other was 1,207,433 bp long in length. The average GC content of the two chromosomes was 57 %. Unsurprisingly, The S2 genomes exhibited significant similarities in size and structure compared to the genomic sequences of 1330. The S2 genome sequences showed over 99.9 % similarities with the genomes of 1330. The diagram of S2 genomes is shown in Fig. 1. Circos was employed to construct the diagram [15]. A total of 2119 and 1139 open reading frames (ORFs) were identified on chromosome I and II, respectively. Sequences of these two chromosomes can be accessed from GenBank (CP006961 and CP006962). More than 99.5 % of ORFs in S2 were identical to those found in 1330 (CP002997.1 and CP002998.1). S2 exhibited highly genome-wide collinearity with 1330 (Fig. 2 and Additional file 1: Table S1). More than 38 % of the predicted ORFs in S2 had rpsblast hits to the cluster of orthologous groups (COG) database with an e-value less than 1e-2. A total of 1147 ORFs (35.2 %) were hypothetical proteins (Table 1).

**Table 1:** Genome properties of the newly sequenced genome of *B. suis* strain S2 in comparison with the known genome sequence of *B. suis* strain 1330

	*B. suis* strain 2		*B. suis* 1330	
Feature/Property	ChrI	ChrII	ChrI	ChrII
ORFs	2119	1139	2122	1144
tRNAs	41	14	41	14
rRNAs	8	4	8	4
Size(bp)	2107842	1207433	2107794	1207381
GC(%)	57.21	57.32	57.21	57.32
Average gene length(bp)	836.28	892.23	836.86	894.46
Coding(%)	84.07	84.17	84.20	84.75
Hypothetical proteins	794	353	796	353

Genome-wide comparison between S2 and 1330 genomes identified all the single nucleotide polymorphisms (SNPs), a total of 72 SNPs were identified (Table 2). The SNPs were not equally distributed between the two chromosomes, 42 SNPs (58 %) were located on chromosome I, while the remaining 30 SNPs (42 %) were located on chromosome II. The exact positions of all of the SNPs at the nucleotide level and protein level (if the SNP is within an ORF) are shown in Additional file 2: Table S2. Twenty-two SNPs (31 %) were located in intergenic regions. Fifty SNPs were located within a total of 48 different ORFs (Table 3). Ten were synonymous substitutions; 39 were non-synonymous substitutions; one SNP caused either frameshifting or premature stop in protein coding region.

**Table 2:** Single nucleotide polymorphism comparison between *B. suis* strain S2 and 1330

	S2 nt					
1330 nt	-	A	C	G	T	Total
Chromosome I						
-		0	0	1	1	2
A	1		3	12	1	17
C	1	2		0	9	12
G	0	5	0		0	5
T	0	1	5	0		6
Chromosome II						
-		0	1	1	0	2
A	0		1	3	0	4
C	2	1		1	3	7
G	2	3	1		3	9
T	0	1	7	0		8
Total	7	12	17	18	17	72

**Table 3:** The identified non-identical proteins between attenuated strain S2 and virulent strain 1330

slno	locus^a^	S2 vs. 1330	Residue change	annotation
1	BS1330_I1369	69△		hypothetical protein (found)
2	BS1330_I0234	8▽	FS	IclR family transcriptional regulator (paper)
3	BS1330_I0875	6△		glycosyl hydrolase family protein(found)
4	BS1330_I2007	2▽	FS/PS	outer membrane autotransporter (paper)
5	BS1330_I0045	1 ⊙^b^	H → R	*qox*D (found)
6	BS1330_I0093	1 ⊙^b^	V → A	*acn*A (found)
7	BS1330_I0235	1 ⊙^b^	S → Y	sugar ABC transporter, periplasmic sugar-binding protein (paper)
8	BS1330_I0501	1 ⊙^b^	Q → R	*ppd*K (surface)
9	BS1330_I0635	1 ⊙^b^	D → A	*omp*2b (paper)
10		1 ⊙^b^	R → H	*omp*2b (paper)
11	BS1330_I0712	1 ⊙^b^	Y → H	hypothetical protein (found)
12	BS1330_I0775	1 ⊙^b^	K → T	hypothetical protein (found)
13	BS1330_I0803	1 ⊙^b^	V → A	*nuo*F (paper)
14	BS1330_I0912	1 ⊙^b^	R → Q	penicillin-binding protein 1A (unknown)
15	BS1330_I1054	1 ⊙^b^	H → R	acetyl-CoA hydrolase/transferase family protein (surface)
16	BS1330_I1203	1 ⊙^b^	P → S	serine protease (unknown)
17	BS1330_I1213	1 ⊙^b^	S → G	*rpl*R (found)
18	BS1330_I1329	1 ⊙^b^	R → H	*ded*A family protein (paper)
19	BS1330_I1458	1 ⊙^b^	H → R	*pnc*A (found)
20	BS1330_I1642	1 ⊙^b^	A → V	*glc*B (surface)
21	BS1330_I1655	1 ⊙^b^	V → A	hypothetical protein (found)
22	BS1330_I1684	1 ⊙^b^	L → R	*glm*M (no direct interaction with substrate)
23	BS1330_I1742	1 ⊙^b^	T → A	*pyk* (no direct interaction with substrate)
24	BS1330_I1896	1 ⊙^b^	Y → H	*sdh*A (found)
25	BS1330_I2038	1 ⊙^b^	E → K	GMC family oxidoreductase (surface)
26	BS1330_I2085	1 ⊙^b^	A → V	sensor histidine kinase BvrS (unknown)
27	BS1330_I2149	1 ⊙^b^	A → D	PhoH family protein (found)
28	BS1330_I0973	1⊙^b^	A	IS711, transposase orfA
29	BS1330_I1083	1⊙^b^	P	luciferase family protein
30	BS1330_I1087	1 ⊙^b^	E	*tgt*
31	BS1330_I1302	1 ⊙^b^	I	*cob*N
32	BS1330_I1971	1 ⊙^b^	L	short chain dehydrogenase
33	BS1330_I2107	1 ⊙^b^	I	hypothetical protein
34	BS1330_II0476	78△		pyridine nucleotide-disulfide oxidoreductase family protein (found)
35	BS1330_II0860	16△	FS/PS	*ery*D (paper)
36	BS1330_II0148	1▽	FS	3-hydroxyisobutyrate dehydrogenase (paper)
37	BS1330_II0683	1△		sugar ABC transporter ATPase (found)
38	BS1330_II0999	1▽		amidase (unknown)
39	BS1330_II1074	1▽^b^	PS	IclR family transcriptional regulator (paper)
40	BS1330_II0278	1 ⊙^b^	A → T	*nnr*R (surface)
41	BS1330_II0440	1 ⊙^b^	A → E	*glp*K (surface)
42	BS1330_II0441	1 ⊙^b^	T → A	major facilitator family transporter (found)
43	BS1330_II0533	1 ⊙^b^	P → Q	oligopeptide ABC transporter, periplasmic oligopeptide-binding protein (paper)
44		1 ⊙^b^	S → G	oligopeptide ABC transporter, periplasmic oligopeptide-binding protein (paper)
45	BS1330_II0567	1 ⊙^b^	S → L	*pcs* (paper)
46	BS1330_II0571	1 ⊙^b^	A → P	peptide ABC transporter, periplasmic peptide-binding protein (found)
47	BS1330_II0782	1 ⊙^b^	V → A	hypothetical protein (found)
48	BS1330_II0786	1 ⊙^b^	M → T	*fad*B (found)
49	BS1330_II0805	1 ⊙^b^	E → K	glucuronate isomerase (interface)
50	BS1330_II0844	1 ⊙^b^	N → D	aminotransferase, class III (surface)
51	BS1330_II0911	1 ⊙^b^	W → R	oxidoreductase, molybdopterin-binding (unknown)
52	BS1330_II0980	1 ⊙^b^	S → P	WD repeat-containing protein (unknown)
53	BS1330_II0990	1 ⊙^b^	C → W	hypothetical protein (found)
54	BS1330_II1031	1 ⊙^b^	P → S	*lep*A (unknown)
55	BS1330_II1137	1 ⊙^b^	M → L	*fli*F (paper)
56	BS1330_II0646	1 ⊙^b^	L	branched-chain amino acid ABC transporter, ATP-binding protein
57	BS1330_II0913	1 ⊙^b^	G	outer surface protein
58	BS1330_II0945	1 ⊙^b^	A	branched-chain amino acid ABC transporter, periplasmic amino acid-binding protein
59	BS1330_II1103	1 ⊙^b^	A	hypothetical protein

*Abbreviations and symbols*: ⊙ = bp Difference, ▽ = bp Deletion, △ = bp insertion, *FS* frameshift, *PS* premature stop; paper: see discussion in the paper

^a^Representative locus from 1330 genomes

^b^single nucleotide polymorphism within ORF

Putative protein-encoding genes, tRNA and rRNA in S2 genome were predicted with RAST (http://rast.nmpdr.org). The predicted protein sequences were compared with non-redundant protein database. The protein sequences were also compared with COG and CDD database, the threshold is E < 1e-2.

### Identification of virulence associated differences between the attenuated strain S2 and virulent strain 1330

The pairwise comparison was carried out to identify the non-identical ORFs between S2 and 1330 (Table 3), which provided useful information on the underlying mechanisms for the attenuation of S2.

Most of the different ORFs between 1330 and S2 in chromosome I and II had only 1 bp difference in nucleotide sequence. Six ORFs had 2, 6, 8, 16, 69 and 78 bp insertion or deletion in the genes respectively (Table 3). The insertion or deletion in the gene caused either frameshifting (including premature stop) or residue insertion in the coding protein. The different ORFs in S2 that encoded the same protein sequences in other *Brucella* strains were labeled ‘found’ and omitted in discussions. The different ORFs labeled with ‘surface’ suggested that the different residue was located on the surface of the modeled structure, which were also omitted from the analysis (Table 3).

### Known virulence associated differences

In chromosome I of S2, a 2 bp deletion caused the premature stop of an outer membrane autotransporter OmaA. The autotransporters are secreted proteins that can translocate themselves through the membranes to the cell surface or to the extracellular environment. Bandara et al. studied the function of this *OmaA* [16]. In mice, the *OmaA*-deficient strain provides greater protection against the challenge of 1330 than that of *B. suis* mutant strain VTRS1. The deficient strain can induce a significantly higher level of serum IgG1 and IgG2a antibodies and can be cleared quickly from the mice. Although the OmaA activity is not necessary in the course of the acute phase of infection of *B. suis*, it might still be a critical virulent factor during the chronic phase of the infection. Therefore, we suspected the lack of intact OmaA protein might be the major cause for S2 virulence attenuation.

The second difference between 1330 and S2 was found in the erythritol (*ery*) operon. The erythritol operon contains 4 ORFs (*eryA, eryB, eryC and eryD*) [17]. Erythritol metabolism by *Brucella* has been known to be associated with the abortion in livestock. The high concentration of erythritol in the foetal tissue of the animal (cattle, sheep, goat and pig) might be advantageous for the growth of *Brucella*. On the other hand, erythritol catabolism pathway might play the important physiological roles in bacteria growth. It has been suggested that erythrose 4-phosphate, the precursor for the biosynthesis of aromatic compounds, may be easily obtained from the intermediates of erythritol catabolism. Compared to 1330, a 16 bp insertion caused the premature stop of the *eryD* gene in S2*.* The studies on *B. abortus* S19 and S2308 showed that *eryD* might act as a repressor of erythritol operon [17]. The consequence of *eryC* and *eryD* ORFs deletion in S19 has been studied, which reveals that they are not sufficient or required for virulence in a murine model. No direct correlation between erythritol metabolism and in vivo colonization was found when S19 and 2308 strains were compared with three genetically engineered strains related to *ery* operon [18]. Considering its repressive control on *ery* operon, the lack of *eryD* might cause the uncontrolled erythritol catabolism. Nevertheless, its consequence on virulence attenuation needs to be further explored.

**Fig. 3:**
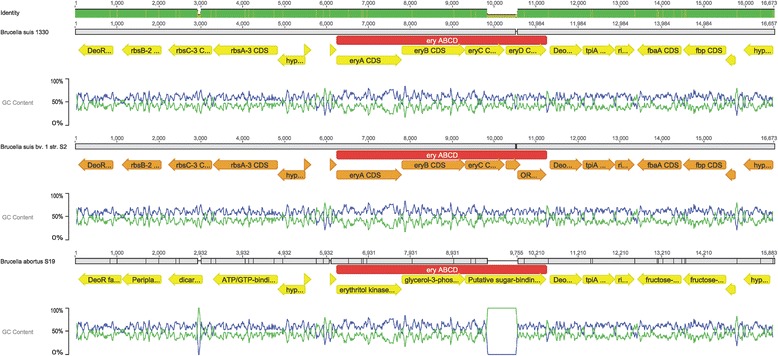
Comparison of the upstream and downstream genomic regions around *ery* operon of *B. suis* strain 1330, *B. suis* strain S2 and *B. abortus* strain S19

It has been reported that S19 does not grow on TSA (Ery). To test the growth capacity of S2 on erythritol, we chose S19 as the control. We found that S2 was able to grow on erythritol agar, suggesting it has the ability to metabolize erythritol (Table 4). We compared the genomes of 1330, S2 and S19 around the *ery* operon. S2 and 1330 genomes have the same *eryA*, *eryB* and *eryC* genes, while *eryD* in S2 genome encodes a premature protein. Meanwhile, a 68 bp deletion in the region of *eryC* and *eryD* results in an ORF encoding a putative sugar binding protein in S19. The other genes upstream and downstream of *ery* operon in these three genomes are highly conserved, except for some mutations in the encoded proteins (Fig. 3). Based on the analysis, the inviability of S19 on erythritol agar might be due to the lack of *eryC* and *eryD* genes in the genome. A recent paper describes a new pathway on erythritol metabolism. Except for the known *eryA*, *eryB*, *eryC* and *eryD* genes, two critical genes downstream of the *eryD gene*, *TpiA2* and *RpiB*, in erythritol metabolism are demonstrated. The new model strongly suggests that erythritol metabolism needs three isomerization reactions sequentially catalyzed by EryC, TpiA2, and RpiB to produce D-erythrose-4-P, which will be converted into glyceraldehyde-3-P and fructose-6-P in the pentose phosphate pathway [19]. This new model clarifies why S19 could not grow on erythritol agar since functional *eryC* is lost in the genome.

**Table 4:** Effect of erythritol on growth of *Brucella suis* strain S2 and *B. abortus* strain S19

Strain	TSA							TSA (Ery)						
	10^7^	10^6^	10^5^	10^4^	10^3^	10^2^	10^1^	10^7^	10^6^	10^5^	10^4^	10^3^	10^2^	10^1^
S19	C	C	+++	+++	++	+	±	±	—	—	—	—	—	—
S2	C	C	+++	+++	+++	++	++	C	C	+++	+++	+++	++	++

Estimate of growth: C, confluent; +++, numerous individual colonies; ++, more than 100 colonies; +, 10 to 100 colonies; ±, 1 to 10 colonies

Phosphatidylcholine synthase gene (*PCS*) in chromosome 2 of S2 strain had one different residue (S32L) compared with PCS protein in 1330. In bacteria, phosphatidylcholine can be synthesized through either the phospholipid N-methylation pathway or the phosphatidylcholine synthase pathway [20, 21]. The correlation between bacterial virulence and membrane phosphatidylcholine has been reported in *L. pneumophila* and *A. tumefaciens* [22–24]. The cell envelope of the *Brucella* contains phosphatidylethanolamine and phosphatidylcholine. Although the phosphatidylcholine level in bacterial membrane does not affect some major virulence properties of *Brucella* such as invasion, intracellular traffic, and intracellular replication, it is necessary to maintain a chronic infection in mice [25, 26]. A systematic study on phosphatidylcholine synthase from *S. meliloti* was performed by alanine scanning mutagenesis. Protein sequence alignment of PCSs from several bacteria species showed that only five of eight residues at position 32 are serine. Changing the conserved residues close to position 32 causes about 20 % decrease in PCS activity [27]. Further studies are needed to explore if the mutation at position 32 affects the PCS activity, which might decrease S2 virulence.

Peptidoglycan is a major component of the bacterial cell wall. Its main function is to preserve the cell integrity and resist osmotic pressure [28]. It has been shown that peptidoglycan synthesis genes affect bacterial shape, elongation, and cell division [29]. Therefore, disruption of peptidoglycan synthesis might affect the survival rate of the bacteria in the host. UDP-N-acetylglucosamine (UDP-GlcNAc) is one of the key building blocks in peptidoglycan biosynthesis [30]. The second step in the formation of UDP-GlcNAc is the interconversion of glucosamine-6-phosphate to glucosamine-1-phosphate, which is catalyzed by the enzyme phosphorglucosamine mutase (PNGM). A growing body of research suggests the importance of PNGM in bacterial virulence and infectivity [31–33]. The different residue in PNGM between S2 and 1330 was located at position 250. Although the residue was close to the substrate binding site, it did not interact with the substrate. The biochemical studies are needed to fully define the function of this residue on PNGM activity and bacterial virulence.

Pyruvate kinase (PK), the key enzyme in the glycolysis, is important for controlling the concentrations of glycolytic intermediates, biosynthetic precursors and nucleoside triphosphates in the cells. Pyruvate kinase transfers the phosphoryl group of phosphoenolpyruvate to ADP to yield pyruvate and ATP. Most eukaryotic PKs can be allosterically activated by fructose 1,6-bisphosphate (FBP), while bacterial PK could be activated by either FBP or AMP/sugar monophosphates [34]. The bacteria carrying the impaired PK activity might exhibit severe attenuation as suggested in the study in *C. Neoformans* [35]. In chromosome I of S2, there was one residue different (T462A) in PK compared to that in 1330. Based on result of the Protein Databank search by blastp program, *Brucella* PK shared some similarities with PKs from other species, such as human, rabbit, *B. Stearothermophilus*, S*. aureus* et al. In the structure of human pyruvate kinase isozyme M2, the corresponding threonine at position 462 was close to the FBP binding site and interacted with FBP through two water molecules [36]. The threonine residue is highly conserved in many PKs, except for the serine in *E. coli*. Both threonine and serine contain hydroxyl groups, which might be important for its interaction with the activator. A study demonstrates that S2 grows normally in the medium containing glucose as the only carbon source [37]. We, therefore, suspected the mutant on PK might not be a major reason for S2 attenuation. Further biochemical studies are still necessary to clarify this conclusion.

Although *Brucella* spp. is considered typically as non-motile, a study demonstrates that flagellar genes are necessary for the assembly of a functional flagellum [38, 39]. *B. melitensis* with *fli*F mutant is unable to replicate in macrophages and HeLa cells, suggesting the *Brucella* flagellar genes might be related to virulence [40]. Another study shows that in the early log phase of its growth in 2YT nutrient broth, *B. melitensis* expresses genes corresponding to the basal (MS ring) and the distal parts of the flagellar apparatus. A polar and sheathed flagellar structure has been observed under transmission electron microscopy. Although the mutations encoding different parts of the flagellum structure do not show a discernible phenotype when compared with the wild-type strain in cellular infection models, all these mutants are attenuated in mice infected via the intraperitoneal route in 4 weeks after infection [41]. We found a mutation in fliF protein (M544L) located in MS ring. Whether the mutation decreases the virulence of S2 awaits further studies.

### Other different proteins found between S2 and 1330

Two *IclR* regulatory genes in S2 were different from those in 1330. The *IclR* regulatory gene located in chromosome I lacked 8 bp that began at ATG, causing the loss of the coding protein. The other different *IclR* regulatory gene located in chromosome II had a 1 bp deletion, resulting in the premature stop in the coding protein. Members of the IclR transcription regulator family regulate a variety of metabolic processes in various microorganisms [42]. In one study, *B. melitensis* 16M mutations in *AraC*, *ArsR*, *Crp*, *DeoR*, *GntR*, *IclR*, *LysR*, *MerR*, *RpiR* and *TetR* were constructed. *GntR* and *LysR* mutants showed the decreased virulence [43]. Two different *IclR* regulatory genes found in S2 genome were correspondent to *IclR5* and *IclR6* in the study. Therefore, the lack of these two *IclR* regulatory genes might not be the major cause for S2 attenuation.

In chromosome I of S2, Omp2b had two residues different from that in 1330. Omp2b is bacterial porins present in the outer membrane of *Brucella*. The differences in the Omp2b variants are located in the predicted external loops of the porin [44]. The first different residue (D79A) is highly conserved in different *Brucella* strains, while the second different residue (R225H) is not strictly conserved. *Omp2b* is present in virulent *B. abortus*, *B. melitensis* and *B. suis* strains that are pathogenic to humans, but is absent in non-pathogenic *B. ovis* [45]. Bacterial porins have been reported to be involved in apoptosis modulation [46–48]. Expression of *B. melitensis* Omp2b in yeast prevents the cell from death induced by the mammalian pro-apoptotic protein Bax [49]. No further functional studies have been reported on *Brucella* Omp2b.

High-affinity binding protein-dependent ABC transporter consists of a high-affinity periplasmic substrate-binding subunit, two hydrophobic membrane subunits and two additional cytoplasmic subunits. ATP hydrolysis by associated ATPase provides the energy for substrate accumulation across the inner membrane against the concentration gradient. Periplasmic substrate-binding subunits are composed of two separate but similarly folded globular domains, which are connected by a hinge region made of two or three short polypeptide segments [50]. In S2 chromosome I, 1 bp difference in periplasmic sugar-binding protein caused one residue difference (S97Y) in protein sequence compared with that in 1330 strain. The sequence alignment with structure-solute receptor GacH of *Streptomyces glaucescens* (Protein Databank accession number: 3JZJ [51]) showed that this residue was located very close to the substrate binding site. Due to the bulky structure of tyrosine, we suspected the mutation might affect the sugar binding affinity or preference of the protein. The comparative proteome study on *B. melitensis* vaccine strain Rev 1 and virulent strain 16 M reveals that four sugar-binding proteins are differentially expressed in Rev 1, although these two strains have similar physical properties in biotyping tests [52]. Therefore, it is possible that the substrate preference change might help bacteria adapt to different growth conditions.

In S2 chromosome II, 2 bp differences resulted in 2 residues difference in oligopeptide ABC transporter. The first different residue (S136G) existed in other *Brucella* strains, while the other different residue was only seen in S2 (P284Q). Bacteria utilize peptides as a source of amino acids, carbon, nitrogen, and/or energy. A variety of peptide uptake systems mediate the translocation of peptides across the bacterial cytoplasmic membrane. Recent studies show that OppA possesses preference in substrate peptide. *E. coli* OppA prefers positively charged peptides with three or four amino acids in length. However, other studies also suggest that the substrate preference of the peptide uptake systems are more complicated [53, 54]. The sequence alignment with the homologous structure of oligopeptide-binding protein from *Salmonella typhimurium* [55] showed that residue 284 was located close to the substrate binding site, but not involved in direct interaction with the substrate. Compared with other available structures of oligopeptide-binding proteins, the residue 284 of OppA protein can be glutamate *(S. typhimurium)*, serine (*E. coli*), alanine (*E. coli*) or tyrosine (*B. Pseudomallei*). Meanwhile, two oligopeptide-binding proteins were encoded in 1330 genome, which shared 84 % similarity in protein sequences. The residue at 284 is aspartate in the other sequence, suggesting this residue was not conserved well in different bacteria. ATP-Binding Cassette Transporter in *Brucella ovis* is shown to be essential in mice [56]. The Δ*abc*A*B* mutant strain can trigger host serologic responses similar to the WT strain and a significant cellular host response [57].

One residue different in S2 was found in a DedA family protein. The DedA family proteins are integral inner membrane proteins, which are present in nearly all species of bacteria. *E. coli* encodes 8 DedA proteins. Although each of them is nonessential, they are collectively essential [58]. An *E. coli* mutant with two *dedA* genes deletion (Δ*yghB*/Δ*yqjA*) fails to complete cell division or grow at elevated temperatures [59]. *B. burgdorferi* possesses only one *dedA* gene. The gene deletion results in imbalanced membrane phospholipid composition, which is required for proper cell division and envelope integrity [60]. No functional clue was suggested for *Brucella DedA* so far.

We identified a difference at residue 576 between S2 and 1330 in a putative molybdopterin-binding oxidoreductase. Members of the molybdopterin oxidoreductase family include formate dehydrogenase, nitrate reductase, DMSO reductase, TMAO reductase, pyrogallol hydroxytransferase and arsenate reductase. The protein in S2 showed about 40 % similarity with formate dehydrogenase H from *E. coli* at residues 54–420, as suggested by the protein databank search with blastp. The residues involved in Mo binding are conserved [61]. No significant sequence similarity was found between these two proteins around residue 576.

A 1 bp insertion caused the premature stop of 3-hydroxyisobutyrate dehydrogenase in S2. 3-hydroxyisobutyrate dehydrogenase, a key enzyme for the metabolism of valine and some keto-bodies, exists widely in bacteria, yeast, and mammalian tissue. It catalyzes the reversible conversion of 3-hydroxyisobutyrate to methylmalonate semialdehyde [62]. Although the enzymatic properties of 3-hydroxyisobutyrate dehydrogenase have been studies in several organisms, its relevance to bacterial virulence awaits further studies.

NADH-quinone oxidoreductase subunit 1 (NuoF) in S2 had one different residue compared with that of 1330 strain. NuoF is part of the hydrophilic fragment of NADH dehydrogenase I, which represents the electron input part of the enzyme NADH: ubiquinone oxidoreductase. It couples electron transport to proton translocation across the membrane, which is partially responsible for generating the proton gradient necessary for ATP production. NADH: ubiquinone oxidoreductase serves as both a proton pump and an entry point for electrons into the respiratory chain [63]. Currently, no bacterial virulence is associated with this gene.

One residue difference was found in *lepA* gene between S2 and 1330. LepA protein encoded by this gene exists in most of the bacterial genomes [64]. Although *lepA* knockout strain of *E. coli* [65] and *Staphylococcus aureus* [66] are viable, the gene product might be necessary for bacteria survival under certain growth conditions [67].

There are other different proteins between S2 and 1330 (labeled unknown in Table 3). When we analyzed the sequences, we did not find any functional implications of the proteins in bacteria. Therefore, further studies are needed to clarify their links to virulence attenuation of S2 strain.

## Conclusion

In this report, we sequenced the attenuated strain *Brucella suis* S2 genome and performed comparative genome analysis between S2 and virulent strain 1330. During the analysis, we found out 59 different ORFs between these two strains. Of these different ORFs, several have been reported to be related to *Brucella* virulence, such as outer membrane autotransporter, eryD. The proteins encoded by some of the different genes have been suggested to be essential for bacteria growth, such as dedA, phosphorglucosamine mutase and phosphatidylcholine synthase. These gene products might be responsible for the utilization of the materials from the environment, which are necessary for bacteria growth. We found several mutations in peptide-binding ATP transporter and sugar-binding ATP transporter, but their roles on S2 attenuation could not be ruled out without further investigation.

It has been shown that the high concentration of erythritol in the foetal tissues of cattle, sheep, goats and pigs might be beneficial for the growth of *Brucella*. Erythritol catabolism pathway may play important physiological roles in *Brucella*. The studies in S19 and S2308 showed that *eryD* might act as the repressor of erythritol operon [17]. In our experiment, we found that S2 but not S19 could grow in the media supplemented with erythritol, suggesting S2 has the ability to metabolize erythritol. Therefore, further investigation on the function of the *eryD* gene is necessary to clarify its relevance to virulence attenuation.

Although we identified some target proteins that might be related to the virulence, most of the identified proteins have not been investigated in *Brucella*. Therefore, all these genes have to be examined individually to ensure their relationships with the virulence. The modification of the genes identified in our study will help to reduce residual virulence, which is critical to developing more efficient *Brucella* vaccine strains.

## Methods

### Strain and genomic DNA preparation

The S2 used in sequencing was obtained from the China Institute of Veterinary Drugs Control. Total genomic DNA was extracted using the DNeasy Blood and Tissue Kit (QIAGEN, China Ltd., China). The DNA was fragmented by nebulization, and fragments ranging from 400 to 600 bp were extracted from an agarose gel after size-fractionation, and then adaptors were added according to Illumina Sample Preparation Guide.

### Genome sequencing and assembly

After sequencing with Illumina Hiseq2000, 50,941,654 paired-end (2*101 bp) reads were obtained. Base calling was performed with the software CAVASA. There were 41,608,314 paired reads passed the filter, which provided coverage of chromosome of ~1000-fold. Reads were assembled into contigs and scaffolds using SOAP de-novo (Release 1.04) [68]. Assembled contigs were compared to the published genome sequence of *B. suis* strain 1330 (NC_017250.1, NC_017251.1) using BLAT v.34 [69]. The order and the orientation of the assembled contigs were determined in accordance with the control genome. Based on the extensive similarities between the published genomes of *Brucella*, PCR primers were designed to link the gaps between two neighboring contigs. The leftover gaps were sequenced separately by conventional Sanger sequencing. The Illumina sequence reads for the genomes are deposited to NCBI, with the accession number of SAMN03068300.

### Gene prediction and annotation

Gene prediction was established with Glimmer 2.0 using the default settings [70]. All the predicted CDS and putative intergenic sequences were subjected to further manual inspections. Exhaustive BLAST searches with an incremental stringency against the NCBI non-redundant protein database were performed to determine the homology of the predicted coding sequences. Translational start codons were identified based on protein homology, proximity to the ribosome-binding site, relative positions to predicted signal peptide and putative promoter sequences. We also classified the putative proteins according to the COG database search results. Transfer RNAs were predicted with the tRNAscan-SE software. The repeat sequences were predicted by Trf program [71]. The results showed 55 and 23 tandem sequences in chromosome I and II. The average copy number is 2.79 and 2.86.

All the S2 proteins predicted have been compared to KEGG bacterial gene set with KAAS (KEGG Automatic Annotation Server http://www.genome.jp/tools/kaas/) program by BBH (Bi-directional best hit) method to map the genes into bacterial pathways automatically.

### Comparative genomic analysis

MUMmer 3.0 program was used to investigate the differences between the genome sequences of 1330 and S2 with the default parameters [72]. The genomes were first automatically compared for SNPs and indels by MUMmer3.0. Then SNPs between 1330 and S2 were called by home-brew perl scripts. We classified SNPs as intergenic, frameshift and premature stop according to their positions and functions, and also counted the numbers of synonymous substitutions and non-synonymous substitutions. To identify potential genes that were different between 1330 and S2, we compared S2 genome sequences using blastp program with proteins download from NCBI RefSeq database.

### S2 and S19 erythritol sensitivity test

The Trypticase-soy agar plates in the presence of 1 mg/ml erythritol [TSA (Ery)] plates were prepared [73]. The autoclaved TSA was cooled to 47 °C, and the appropriate amount of erythritol was added and mixed. The plates were poured. S2 and S19 were grown on TSA slants at 37 °C for 24 h and resuspended in saline, and were tested by placing 20 μl of tenfold dilutions (10^7^, 10^6^, 10^5^, 10^4^, 10^3^, 10^2^ and 10^1^ CFU/ml) of the bacteria resuspension on TSA and TSA (Ery) plates and incubated at 37 °C. Plates were examined for 2 to 5 days. The effect of erythritol on the growth of S2 was estimated from the size of the colonies on the test plates in comparison with the control plates (S2 and S19 inoculated on TSA plates and S19 inoculated on TSA (Ery) plates). The experiments were repeated three times.

## Additional files

Additional file 1: Table S1. Results of genome-wide collinear analysis between attenuated strain S2 and virulent strain 1330 by Mummer. (DOC 56 kb) Additional file 2: Table S2. Single nucleotide polymorphism difference between attenuated strain S2 and virulent strain 1330. (DOC 146 kb)

## References

[1] Pappas G, Papadimitriou P, Akritidis N, Christou L, Tsianos EV (2006). The new global map of human brucellosis. Lancet Infect Dis.

[2] O'Callaghan D, Whatmore AM (2011). Brucella genomics as we enter the multi-genome era. Brief Funct Genomics.

[3] DelVecchio VG, Kapatral V, Redkar RJ, Patra G, Mujer C, Los T, Ivanova N, Anderson I, Bhattacharyya A, Lykidis A (2002). The genome sequence of the facultative intracellular pathogen Brucella melitensis. Proc Natl Acad Sci U S A.

[4] Paulsen IT, Seshadri R, Nelson KE, Eisen JA, Heidelberg JF, Read TD, Dodson RJ, Umayam L, Brinkac LM, Beanan MJ (2002). The Brucella suis genome reveals fundamental similarities between animal and plant pathogens and symbionts. Proc Natl Acad Sci U S A.

[5] Tae H, Shallom S, Settlage R, Preston D, Adams LG, Garner HR (2011). Revised genome sequence of Brucella suis 1330. J Bacteriol.

[6] Crasta OR, Folkerts O, Fei Z, Mane SP, Evans C, Martino-Catt S, Bricker B, Yu G, Du L, Sobral BW (2008). Genome sequence of Brucella abortus vaccine strain S19 compared to virulent strains yields candidate virulence genes. PLoS One.

[7] Morgan WJ, Corbel MJ (1976). Recommendations for the description of species and biotypes of the genus Brucella. Dev Biol Stand.

[8] Moreno E, Cloeckaert A, Moriyon I (2002). Brucella evolution and taxonomy. Vet Microbiol.

[9] Xin X (1986). Orally administrable brucellosis vaccine: Brucella suis strain 2 vaccine. Vaccine.

[10] Deqiu S, Donglou X, Jiming Y (2002). Epidemiology and control of brucellosis in China. Vet Microbiol.

[11] Verger JM, Grayon M, Zundel E, Lechopier P, Olivier-Bernardin V (1995). Comparison of the efficacy of Brucella suis strain 2 and Brucella melitensis Rev. 1 live vaccines against a Brucella melitensis experimental infection in pregnant ewes. Vaccine.

[12] Blasco JM, Marin C, De Bagues Jimenez MP, Barberan M (1993). Efficacy of Brucella suis strain 2 vaccine against Brucella ovis in rams. Vaccine.

[13] Mustafa AA, Abusowa M (1993). Field-oriented trial of the Chinese Brucella suis strain 2 vaccine on sheep and goats in Libya. Vet Res.

[14] Bosseray N, Plommet M (1990). Brucella suis S2, brucella melitensis Rev. 1 and Brucella abortus S19 living vaccines: residual virulence and immunity induced against three Brucella species challenge strains in mice. Vaccine.

[15] Krzywinski M, Schein J, Birol I, Connors J, Gascoyne R, Horsman D, Jones SJ, Marra MA (2009). Circos: an information aesthetic for comparative genomics. Genome Res.

[16] Bandara AB, Sriranganathan N, Schurig GG, Boyle SM (2005). Putative outer membrane autotransporter protein influences survival of Brucella suis in BALB/c mice. Vet Microbiol.

[17] Sangari FJ, Aguero J, Garcia-Lobo JM (2000). The genes for erythritol catabolism are organized as an inducible operon in Brucella abortus. Microbiology.

[18] Sangari FJ, Grillo MJ, De Bagues Jimenez MP, Gonzalez-Carrero MI, Garcia-Lobo JM, Blasco JM, Aguero J (1998). The defect in the metabolism of erythritol of the Brucella abortus B19 vaccine strain is unrelated with its attenuated virulence in mice. Vaccine.

[19] Barbier T, Collard F, Zuniga-Ripa A, Moriyon I, Godard T, Becker J, Wittmann C, Van Schaftingen E, Letesson JJ (2014). Erythritol feeds the pentose phosphate pathway via three new isomerases leading to D-erythrose-4-phosphate in Brucella. Proc Natl Acad Sci U S A.

[20] Sohlenkamp C, de Rudder KE, Rohrs V, Lopez-Lara IM, Geiger O (2000). Cloning and characterization of the gene for phosphatidylcholine synthase. J Biol Chem.

[21] de Rudder KE, Sohlenkamp C, Geiger O (1999). Plant-exuded choline is used for rhizobial membrane lipid biosynthesis by phosphatidylcholine synthase. J Biol Chem.

[22] Klusener S, Aktas M, Thormann KM, Wessel M, Narberhaus F (2009). Expression and physiological relevance of Agrobacterium tumefaciens phosphatidylcholine biosynthesis genes. J Bacteriol.

[23] Conover GM, Martinez-Morales F, Heidtman MI, Luo ZQ, Tang M, Chen C, Geiger O, Isberg RR (2008). Phosphatidylcholine synthesis is required for optimal function of Legionella pneumophila virulence determinants. Cell Microbiol.

[24] Wessel M, Klusener S, Godeke J, Fritz C, Hacker S, Narberhaus F (2006). Virulence of Agrobacterium tumefaciens requires phosphatidylcholine in the bacterial membrane. Mol Microbiol.

[25] Comerci DJ, Altabe S, de Mendoza D, Ugalde RA (2006). Brucella abortus synthesizes phosphatidylcholine from choline provided by the host. J Bacteriol.

[26] Conde-Alvarez R, Grillo MJ, Salcedo SP, de Miguel MJ, Fugier E, Gorvel JP, Moriyon I, Iriarte M (2006). Synthesis of phosphatidylcholine, a typical eukaryotic phospholipid, is necessary for full virulence of the intracellular bacterial parasite Brucella abortus. Cell Microbiol.

[27] Solis-Oviedo RL, Martinez-Morales F, Geiger O, Sohlenkamp C (2012). Functional and topological analysis of phosphatidylcholine synthase from Sinorhizobium meliloti. Biochim Biophys Acta.

[28] van Heijenoort J (2001). Formation of the glycan chains in the synthesis of bacterial peptidoglycan. Glycobiology.

[29] Cloud KA, Dillard JP (2002). A lytic transglycosylase of Neisseria gonorrhoeae is involved in peptidoglycan-derived cytotoxin production. Infect Immun.

[30] Barreteau H, Kovac A, Boniface A, Sova M, Gobec S, Blanot D (2008). Cytoplasmic steps of peptidoglycan biosynthesis. FEMS Microbiol Rev.

[31] Yajima A, Takahashi Y, Shimazu K, Urano-Tashiro Y, Uchikawa Y, Karibe H, Konishi K (2009). Contribution of phosphoglucosamine mutase to the resistance of Streptococcus gordonii DL1 to polymorphonuclear leukocyte killing. FEMS Microbiol Lett.

[32] Shimazu K, Takahashi Y, Uchikawa Y, Shimazu Y, Yajima A, Takashima E, Aoba T, Konishi K (2008). Identification of the Streptococcus gordonii glmM gene encoding phosphoglucosamine mutase and its role in bacterial cell morphology, biofilm formation, and sensitivity to antibiotics. FEMS Immunol Med Microbiol.

[33] Liu XD, Duan J, Guo LH (2009). Role of phosphoglucosamine mutase on virulence properties of Streptococcus mutans. Oral Microbiol Immunol.

[34] Johnsen U, Hansen T, Schonheit P (2003). Comparative analysis of pyruvate kinases from the hyperthermophilic archaea Archaeoglobus fulgidus, Aeropyrum pernix, and Pyrobaculum aerophilum and the hyperthermophilic bacterium Thermotoga maritima: unusual regulatory properties in hyperthermophilic archaea. J Biol Chem.

[35] Price MS, Betancourt-Quiroz M, Price JL, Toffaletti DL, Vora H, Hu G, Kronstad JW, Perfect JR (2011). Cryptococcus neoformans requires a functional glycolytic pathway for disease but not persistence in the host. mBio.

[36] Dombrauckas JD, Santarsiero BD, Mesecar AD (2005). Structural basis for tumor pyruvate kinase M2 allosteric regulation and catalysis. Biochemistry.

[37] Plommet M (1991). Minimal requirements for growth of Brucella suis and other Brucella species. Zentralblatt fur Bakteriologie: Int J Med Microbiol.

[38] Sanchez DO, Zandomeni RO, Cravero S, Verdun RE, Pierrou E, Faccio P, Diaz G, Lanzavecchia S, Aguero F, Frasch AC (2001). Gene discovery through genomic sequencing of Brucella abortus. Infect Immun.

[39] Halling SM (1998). On the presence and organization of open reading frames of the nonmotile pathogen Brucella abortus similar to class II, III, and IV flagellar genes and to LcrD virulence superfamily. Microb Comp Genomics.

[40] Lestrate P, Dricot A, Delrue RM, Lambert C, Martinelli V, De Bolle X, Letesson JJ, Tibor A (2003). Attenuated signature-tagged mutagenesis mutants of Brucella melitensis identified during the acute phase of infection in mice. Infect Immun.

[41] Fretin D, Fauconnier A, Kohler S, Halling S, Leonard S, Nijskens C, Ferooz J, Lestrate P, Delrue RM, Danese I (2005). The sheathed flagellum of Brucella melitensis is involved in persistence in a murine model of infection. Cell Microbiol.

[42] Molina-Henares AJ, Krell T, Eugenia Guazzaroni M, Segura A, Ramos JL (2006). Members of the IclR family of bacterial transcriptional regulators function as activators and/or repressors. FEMS Microbiol Rev.

[43] Haine V, Sinon A, Van Steen F, Rousseau S, Dozot M, Lestrate P, Lambert C, Letesson JJ, De Bolle X (2005). Systematic targeted mutagenesis of Brucella melitensis 16M reveals a major role for GntR regulators in the control of virulence. Infect Immun.

[44] Paquet JY, Diaz MA, Genevrois S, Grayon M, Verger JM, de Bolle X, Lakey JH, Letesson JJ, Cloeckaert A (2001). Molecular, antigenic, and functional analyses of Omp2b porin size variants of Brucella spp. J Bacteriol.

[45] He Y, Xiang Z (2010). Bioinformatics analysis of Brucella vaccines and vaccine targets using VIOLIN. Immunome Research.

[46] Kozjak-Pavlovic V, Dian-Lothrop EA, Meinecke M, Kepp O, Ross K, Rajalingam K, Harsman A, Hauf E, Brinkmann V, Gunther D (2009). Bacterial porin disrupts mitochondrial membrane potential and sensitizes host cells to apoptosis. PLoS Pathog.

[47] Massari P, King CA, Ho AY, Wetzler LM (2003). Neisserial PorB is translocated to the mitochondria of HeLa cells infected with Neisseria meningitidis and protects cells from apoptosis. Cell Microbiol.

[48] Muller A, Gunther D, Dux F, Naumann M, Meyer TF, Rudel T (1999). Neisserial porin (PorB) causes rapid calcium influx in target cells and induces apoptosis by the activation of cysteine proteases. EMBO J.

[49] Laloux G, Deghelt M, de Barsy M, Letesson JJ, De Bolle X (2010). Identification of the essential Brucella melitensis porin Omp2b as a suppressor of Bax-induced cell death in yeast in a genome-wide screening. PLoS One.

[50] Diez J, Diederichs K, Greller G, Horlacher R, Boos W, Welte W (2001). The crystal structure of a liganded trehalose/maltose-binding protein from the hyperthermophilic Archaeon Thermococcus litoralis at 1.85 A. J Mol Biol.

[51] Vahedi-Faridi A, Licht A, Bulut H, Scheffel F, Keller S, Wehmeier UF, Saenger W, Schneider E (2010). Crystal structures of the solute receptor GacH of Streptomyces glaucescens in complex with acarbose and an acarbose homolog: comparison with the acarbose-loaded maltose-binding protein of Salmonella typhimurium. J Mol Biol.

[52] Eschenbrenner M, Wagner MA, Horn TA, Kraycer JA, Mujer CV, Hagius S, Elzer P, DelVecchio VG (2002). Comparative proteome analysis of Brucella melitensis vaccine strain Rev 1 and a virulent strain, 16M. J Bacteriol.

[53] Klepsch MM, Kovermann M, Low C, Balbach J, Permentier HP, Fusetti F, de Gier JW, Slotboom DJ, Berntsson RP (2011). Escherichia coli peptide binding protein OppA has a preference for positively charged peptides. J Mol Biol.

[54] Berntsson RP, Doeven MK, Fusetti F, Duurkens RH, Sengupta D, Marrink SJ, Thunnissen AM, Poolman B, Slotboom DJ (2009). The structural basis for peptide selection by the transport receptor OppA. EMBO J.

[55] Tame JR, Murshudov GN, Dodson EJ, Neil TK, Dodson GG, Higgins CF, Wilkinson AJ (1994). The structural basis of sequence-independent peptide binding by OppA protein. Science.

[56] Silva TM, Paixao TA, Costa EA, Xavier MN, Sa JC, Moustacas VS, den Hartigh AB, Carvalho Neta AV, Oliveira SC, Tsolis R (2011). Putative ATP-binding cassette transporter is essential for Brucella ovis pathogenesis in mice. Infect Immun.

[57] Silva AP, Macedo AA, Costa LF, Turchetti AP, Bull V, Pessoa MS, Araujo MS, Nascimento EF, Martins-Filho OA, Paixao TA (2013). Brucella ovis lacking a species-specific putative ATP-binding cassette transporter is attenuated but immunogenic in rams. Vet Microbiol.

[58] Boughner LA, Doerrler WT (2012). Multiple deletions reveal the essentiality of the DedA membrane protein family in Escherichia coli. Microbiology.

[59] Thompkins K, Chattopadhyay B, Xiao Y, Henk MC, Doerrler WT (2008). Temperature sensitivity and cell division defects in an Escherichia coli strain with mutations in yghB and yqjA, encoding related and conserved inner membrane proteins. J Bacteriol.

[60] Liang FT, Xu Q, Sikdar R, Xiao Y, Cox JS, Doerrler WT (2010). BB0250 of Borrelia burgdorferi is a conserved and essential inner membrane protein required for cell division. J Bacteriol.

[61] Boyington JC, Gladyshev VN, Khangulov SV, Stadtman TC, Sun PD (1997). Crystal structure of formate dehydrogenase H: catalysis involving Mo, molybdopterin, selenocysteine, and an Fe4S4 cluster. Science.

[62] Bannerjee D, Sanders LE, Sokatch JR (1970). Properties of purified methylmalonate semialdehyde dehydrogenase of Pseudomonas aeruginosa. J Biol Chem.

[63] Braun M, Bungert S, Friedrich T (1998). Characterization of the overproduced NADH dehydrogenase fragment of the NADH:ubiquinone oxidoreductase (complex I) from Escherichia coli. Biochemistry.

[64] Margus T, Remm M, Tenson T (2007). Phylogenetic distribution of translational GTPases in bacteria. BMC Genomics.

[65] Dibb NJ, Wolfe PB (1986). lep operon proximal gene is not required for growth or secretion by Escherichia coli. J Bacteriol.

[66] Colca JR, McDonald WG, Waldon DJ, Thomasco LM, Gadwood RC, Lund ET, Cavey GS, Mathews WR, Adams LD, Cecil ET (2003). Cross-linking in the living cell locates the site of action of oxazolidinone antibiotics. J Biol Chem.

[67] Bijlsma JJ, Lie ALM, Nootenboom IC, Vandenbroucke-Grauls CM, Kusters JG (2000). Identification of loci essential for the growth of Helicobacter pylori under acidic conditions. J Infect Dis.

[68] Li R, Zhu H, Ruan J, Qian W, Fang X, Shi Z, Li Y, Li S, Shan G, Kristiansen K (2010). De novo assembly of human genomes with massively parallel short read sequencing. Genome Res.

[69] Kent WJ (2002). BLAT--the BLAST-like alignment tool. Genome Res.

[70] Delcher AL, Harmon D, Kasif S, White O, Salzberg SL (1999). Improved microbial gene identification with GLIMMER. Nucleic Acids Res.

[71] Benson G (1999). Tandem repeats finder: a program to analyze DNA sequences. Nucleic Acids Res.

[72] Delcher AL, Phillippy A, Carlton J, Salzberg SL (2002). Fast algorithms for large-scale genome alignment and comparison. Nucleic Acids Res.

[73] Jones LM, Montgomery V, Wilson JB (1965). Characteristics of Carbon Dioxide-Independent Cultures of Brucella Abortus Isolated from Cattle Vaccinated with Strain 19. J Infect Dis.

