# Supplementary material for: Comparative genomic analysis between newly sequenced Brucella suis Vaccine Strain S2 and the Virulent Brucella suis Strain 1330
Source: BMC Genomics. 2016 Sep 20;17:741. doi: 10.1186/s12864-016-3076-5 (PMC5029015; doi:10.1186/s12864-016-3076-5)
Supplement: Additional file 1: Table S1. — Results of genome-wide collinear analysis between attenuated strain S2 and virulent strain 1330 by Mummer. (DOC 56 kb) [file 12864_2016_3076_MOESM1_ESM.doc]

Additional file 1: Table S1 Results of genome-wide collinear analysis between *B. suis* strain 2 and strain 1330 by Mummer

| ID | Chromosome | 1330 | | S2 | | Fragment Length | | Identity (%) | Type |
| --- | --- | --- | --- | --- | --- | --- | --- | --- | --- |
|  |  | Start | End | Start | End | 1330 | S2 |  |  |
| RD1 | chr1 | 1 | 62858 | 1 | 62858 | 62858 | 62858 | 99.99 | No Change |
| RD2 | chr1 | 62810 | 64760 | 62866 | 64816 | 1951 | 1951 | 100 | No Change |
| RD3 | chr1 | 64782 | 97044 | 64782 | 97068 | 32263 | 32287 | 99.93 | No Change |
| RD4 | chr1 | 77065 | 448904 | 77065 | 448897 | 371840 | 371833 | 99.98 | No Change |
| RD5 | chr1 | 267508 | 272289 | 1055334 | 1050553 | 4782 | 4782 | 99.77 | Reverse Exchange |
| RD6 | chr1 | 428879 | 746305 | 428880 | 746291 | 317427 | 317412 | 99.99 | No Change |
| RD8 | chr1 | 632593 | 633700 | 631780 | 630658 | 1108 | 1123 | 86.1 | Reverse |
| RD9 | chr1 | 726279 | 864025 | 726273 | 864018 | 137747 | 137746 | 99.99 | No Change |
| RD10 | chr1 | 844033 | 1331643 | 844020 | 1331636 | 487611 | 487617 | 99.99 | No Change |
| RD11 | chr1 | 943798 | 944437 | 1066241 | 1065602 | 640 | 640 | 99.69 | Reverse Exchange |
| RD12 | chr1 | 1050560 | 1055341 | 272290 | 267509 | 4782 | 4782 | 99.77 | Reverse Exchange |
| RD13 | chr1 | 1065609 | 1066248 | 944430 | 943791 | 640 | 640 | 99.84 | Reverse Exchange |
| RD14 | chr1 | 1331629 | 2107783 | 1331691 | 2107842 | 776155 | 776152 | 99.99 | No Change |
| RD16 | chr1 | 1785812 | 1786627 | 1782580 | 1781765 | 816 | 816 | 100 | Reverse |
| RD17 | chr2 | 1 | 73118 | 1 | 73118 | 73118 | 73118 | 100 | No Change |
| RD18 | chr2 | 73110 | 463074 | 73070 | 463032 | 389965 | 389963 | 99.99 | No Change |
| RD19 | chr2 | 463063 | 862939 | 463099 | 862989 | 399877 | 399891 | 99.99 | No Change |
| RD20 | chr2 | 531818 | 532662 | 833385 | 832542 | 845 | 844 | 99.05 | Reverse Exchange |
| RD21 | chr2 | 832508 | 833351 | 532699 | 531855 | 844 | 845 | 99.05 | Reverse Exchange |
| RD22 | chr2 | 842958 | 1207380 | 842992 | 1207433 | 364423 | 364442 | 99.99 | No Change |
| RD23 | chr2 | 1055337 | 1056554 | 1103896 | 1102679 | 1218 | 1218 | 100 | Reverse Exchange |
| RD24 | chr2 | 1102626 | 1103843 | 1056608 | 1055391 | 1218 | 1218 | 100 | Reverse Exchange |

Reverse: the direction of sequence changed; Reverse exchange: the position and the direction changed.
