# Supplementary material for: Comparative genomic analysis between newly sequenced Brucella suis Vaccine Strain S2 and the Virulent Brucella suis Strain 1330
Source: BMC Genomics. 2016 Sep 20;17:741. doi: 10.1186/s12864-016-3076-5 (PMC5029015; doi:10.1186/s12864-016-3076-5)
Supplement: Additional file 2: Table S2. — Single nucleotide polymorphism difference between attenuated strain S2 and virulent strain 1330. (DOC 146 kb) [file 12864_2016_3076_MOESM2_ESM.doc]

Additional file 2: Table S2 Single nucleotide polymorphism difference between *B. suis* strain S2 and strain 1330

|  | Loca of  1330 | | 1330 | | S2 | | IN/DEL | | In/OUT CDS | | CDS | | Strand | | Codon 1330 | | Codon S2 | | AA of 1330 | | AA of S2 | | synonymous/non- synonymous | |
| --- | --- | --- | --- | --- | --- | --- | --- | --- | --- | --- | --- | --- | --- | --- | --- | --- | --- | --- | --- | --- | --- | --- | --- | --- |
| Chr I | 29269 | | A | | G | |  | | out | |  | |  | |  | |  | |  | |  | |  | |
| Chr I | 50509 | | A | | G | |  | | in | | 50157-50522 | | + | | CAC | | CGC | | H | | R | | non- synonymous | |
| Chr I | 98339 | | A | | G | |  | | in | | 97084-99771 | | - | | GTC | | GCC | | V | | A | | non- synonymous | |
| Chr I | 213406 | | A | | . | | DEL | | out | |  | |  | |  | |  | |  | |  | |  | |
| Chr I | 228045 | | G | | A | |  | | out | |  | |  | |  | |  | |  | |  | |  | |
| Chr I | 247396 | | C | | A | |  | | in | | 247107-248426 | | + | | TCT | | TAT | | S | | Y | | non- synonymous | |
| Chr I | 501463 | | A | | G | |  | | in | | 500178-502841 | | + | | CAG | | CGG | | Q | | R | | non- synonymous | |
| Chr I | 632806 | | A | | C | |  | | in | | 632571-633698 | | + | | GAC | | GCC | | D | | A | | non- synonymous | |
| Chr I | 633244 | | G | | A | |  | | in | | 632571-633698 | | + | | CGC | | CAC | | R | | H | | non- synonymous | |
| Chr I | 682516 | | . | | T | | IN | | out | |  | |  | |  | |  | |  | |  | |  | |
| Chr I | 701244 | | A | | G | |  | | in | | 700885-701670 | | - | | TAC | | CAC | | Y | | H | | non- synonymous | |
| Chr I | 704973 | | A | | T | |  | | out | |  | |  | |  | |  | |  | |  | |  | |
| Chr I | 725942 | | T | | A | |  | | out | |  | |  | |  | |  | |  | |  | |  | |
| Chr I | 726138 | | C | | T | |  | | out | |  | |  | |  | |  | |  | |  | |  | |
| Chr I | 763396 | | A | | C | |  | | in | | 763365-763907 | | + | | AAG | | ACG | | K | | T | | non- synonymous | |
| Chr I | 764575 | | . | | G | | IN | | out | |  | |  | |  | |  | |  | |  | |  | |
| Chr I | 783796 | | T | | C | |  | | in | | 782949-784256 | | + | | GTC | | GCC | | V | | A | | non- synonymous | |
| Chr I | 888550 | | G | | A | |  | | in | | 888354-890813 | | + | | CGA | | CAA | | R | | Q | | non- synonymous | |
| Chr I | 944595 | | G | | A | |  | | in | | 944530-944841 | | + | | GCG | | GCA | | A | | A | | synonymous | |
| Chr I | 1027903 | | T | | C | |  | | in | | 1027254-1028744 | | - | | CAC | | CGC | | H | | R | | non- synonymous | |
| Chr I | 1059895 | | A | | G | |  | | in | | 1059553-1060536 | | - | | CCT | | CCC | | P | | P | | synonymous | |
| Chr I | 1066226 | | C | | T | |  | | in | | 1066741-1065608 | | - | | GAG | | GAA | | E | | E | | synonymous | |
| Chr I | 1184823 | G | | A | |  | | in | | 1184482-1185906 | | - | | CCG | | TCG | | P | | S | | non- synonymous | |  |
| Chr I | 1192661 | T | | C | |  | | in | | 1192590-1192952 | | - | | AGT | | GGT | | S | | G | | non- synonymous | |  |
| Chr I | 1264439 | A | | G | |  | | in | | 1264250-1268041 | | - | | ATT | | ATC | | I | | I | | synonymous | |  |
| Chr I | 1296620 | C | | T | |  | | in | | 1296214-1296876 | | - | | CGC | | CAC | | R | | H | | non- synonymous | |  |
| Chr I | 1421871 | A | | G | |  | | in | | 1421540-1422169 | | + | | CAC | | CGC | | H | | R | | non- synonymous | |  |
| Chr I | 1498205 | T | | C | |  | | out | |  | |  | |  | |  | |  | |  | |  | |  |
| Chr I | 1597922 | C | | T | |  | | in | | 1597141-1599327 | | + | | GCG | | GTG | | A | | V | | non- synonymous | |  |
| Chr I | 1601486 | A | | G | |  | | out | |  | |  | |  | |  | |  | |  | |  | |  |
| Chr I | 1609623 | A | | G | |  | | in | | 1609565-1609918 | | - | | GTT | | GCT | | V | | A | | non- synonymous | |  |
| Chr I | 1634008 | A | | C | |  | | in | | 1633401-1634756 | | - | | CTT | | CGT | | L | | R | | non- synonymous | |  |
| Chr I | 1674842 | C | | . | | DEL | | out | |  | |  | |  | |  | |  | |  | |  | |  |
| Chr I | 1674847 | C | | T | |  | | out | |  | |  | |  | |  | |  | |  | |  | |  |
| Chr I | 1682399 | T | | C | |  | | in | | 1682346-1683782 | | - | | ACC | | GCC | | T | | A | | non- synonymous | |  |
| Chr I | 1717732 | C | | T | |  | | out | |  | |  | |  | |  | |  | |  | |  | |  |
| Chr I | 1838564 | A | | G | |  | | in | | 1836783-1838624 | | - | | TAC | | CAC | | Y | | H | | non- synonymous | |  |
| Chr I | 1905058 | C | | T | |  | | in | | 1904704-1905546 | | - | | CTG | | CTA | | L | | L | | synonymous | |  |
| Chr I | 1968985 | C | | T | |  | | in | | 1967798-1969282 | | - | | GAG | | AAG | | E | | K | | non- synonymous | |  |
| Chr I | 2014830 | C | | T | |  | | in | | 2013875-2015680 | | + | | GCT | | GTT | | A | | V | | non- synonymous | |  |
| Chr I | 2037923 | A | | G | |  | | in | | 2037767-2038528 | | - | | ATT | | ATC | | I | | I | | synonymous | |  |
| Chr I | 2075799 | C | | A | |  | | in | | 2075537-2076520 | | + | | GCC | | GAC | | A | | D | | non- synonymous | |  |
| Chr II | 141574 | G | | . | | DEL | | in | | 141127-142029 | | - | | CCC | | CCG | | P | | P | |  | |  |
| Chr II | 265249 | G | | A | |  | | in | | 264895-265593 | | + | | GCA | | ACA | | A | | T | | non- synonymous | |  |
| Chr II | 361462 | C | | . | | DEL | | out | |  | |  | |  | |  | |  | |  | |  | |  |
| Chr II | 431764 | G | | T | |  | | in | | 430458-431954 | | - | | GCA | | GAA | | A | | E | | non- synonymous | |  |
| Chr II | 432146 | T | | C | |  | | in | | 432081-433382 | | - | | ACA | | GCA | | T | | A | | non- synonymous | |  |
| Chr II | 519189 | . | | C | | IN | | out | |  | |  | |  | |  | |  | |  | |  | |  |
| Chr II | 519964 | G | | T | |  | | in | | 519237-520814 | | - | | CCG | | CAG | | P | | Q | | non- synonymous | |  |
| Chr II | 520409 | T | | C | |  | | in | | 519237-520814 | | - | | AGC | | GGC | | S | | G | | non- synonymous | |  |
| Chr II | 520886 | C | | A | |  | | out | |  | |  | |  | |  | |  | |  | |  | |  |
| Chr II | 540176 | C | | G | |  | | out | |  | |  | |  | |  | |  | |  | |  | |  |
| Chr II | 551232 | G | | A | |  | | in | | 550526-551326 | | - | | TCG | | TTG | | S | | L | | non- synonymous | |  |
| Chr II | 555597 | G | | C | |  | | in | | 554406-556253 | | + | | GCC | | CCC | | A | | P | | non- synonymous | |  |
| Chr II | 632939 | T | | C | |  | | in | | 632573-633289 | | + | | TTG | | CTG | | L | | L | | synonymous | |  |
| Chr II | 669245 | . | | G | | IN | | in | | 669006-670120 | | - | | TGA | | CTG | | Stop | | L | |  | |  |
| Chr II | 713593 | G | | T | |  | | out | |  | |  | |  | |  | |  | |  | |  | |  |
| Chr II | 770657 | A | | G | |  | | in | | 770632-771042 | | - | | GTC | | GCC | | V | | A | | non- synonymous | |  |
| Chr II | 776170 | A | | G | |  | | in | | 774834-777050 | | - | | ATG | | ACG | | M | | T | | non- synonymous | |  |
| Chr II | 796689 | C | | T | |  | | in | | 796378-797778 | | - | | GAA | | AAA | | E | | K | | non- synonymous | |  |
| Chr II | 837080 | T | | C | |  | | in | | 836148-837602 | | - | | AAT | | GAT | | N | | D | | non- synonymous | |  |
| Chr II | 903325 | A | | G | |  | | in | | 902738-905050 | | - | | TGG | | CGG | | W | | R | | non- synonymous | |  |
| Chr II | 907485 | C | | T | |  | | in | | 906679-907533 | | + | | GGC | | GGT | | G | | G | | synonymous | |  |
| Chr II | 943835 | T | | C | |  | | in | | 943032-944237 | | + | | GCT | | GCC | | A | | A | | synonymous | |  |
| Chr II | 976058 | T | | C | |  | | in | | 975098-976072 | | + | | TCG | | CCG | | S | | P | | non- synonymous | |  |
| Chr II | 985459 | A | | C | |  | | in | | 985435-986616 | | - | | TGT | | TGG | | C | | W | | non- synonymous | |  |
| Chr II | 996609 | C | | . | | DEL | | in | | 996027-997416 | | - | | GGA | | G-A | | G | | - | |  | |  |
| Chr II | 1025446 | G | | A | |  | | in | | 1024376-1026184 | | - | | CCC | | TCC | | P | | S | | non- synonymous | |  |
| Chr II | 1064663 | G | | . | | DEL | | in | | 1064528-1065334 | | + | | ACG | | AC- | | T | | - | |  | |  |
| Chr II | 1094166 | T | | C | |  | | out | |  | |  | |  | |  | |  | |  | |  | |  |
| Chr II | 1099309 | C | | T | |  | | in | | 1099277-1100536 | | + | | GCC | | GCT | | A | | A | | synonymous | |  |
| Chr II | 1134264 | T | | A | |  | | in | | 1134151-1135893 | | - | | ATG | | TTG | | M | | L | | non- synonymous | |  |
